# Resistance to Carbapenems in Non-Typhoidal *Salmonella enterica* Serovars from Humans, Animals and Food

**DOI:** 10.3390/vetsci5020040

**Published:** 2018-04-08

**Authors:** Javier Fernández, Beatriz Guerra, M. Rosario Rodicio

**Affiliations:** 1 Servicio de Microbiología, Hospital Universitario Central de Asturias, Oviedo 33011, Spain; javifdom@gmail.com; 2 Instituto de Investigación del Principado de Asturias (ISPA), Oviedo 33011, Spain; 3 European Food Safety Authority, Parma 43121, Italy; beatriz.guerra@efsa.europa.eu; 4 Departamento de Biología Funcional, Área de Microbiología, Universidad de Oviedo, Oviedo 33006, Spain

**Keywords:** *Salmonella enterica*, multidrug resistance, carbapenems, carbapenemases, mobile genetic elements, plasmids, porins

## Abstract

Non-typhoidal serovars of *Salmonella enterica* (NTS) are a leading cause of food-borne disease in animals and humans worldwide. Like other zoonotic bacteria, NTS have the potential to act as reservoirs and vehicles for the transmission of antimicrobial drug resistance in different settings. Of particular concern is the resistance to critical “last resort” antimicrobials, such as carbapenems. In contrast to other *Enterobacteriaceae* (e.g., *Klebsiella pneumoniae*, *Escherichia coli*, and *Enterobacter*, which are major nosocomial pathogens affecting debilitated and immunocompromised patients), carbapenem resistance is still very rare in NTS. Nevertheless, it has already been detected in isolates recovered from humans, companion animals, livestock, wild animals, and food. Five carbapenemases with major clinical importance—namely KPC (*Klebsiella pneumoniae* carbapenemase) (class A), IMP (imipenemase), NDM (New Delhi metallo-β-lactamase), VIM (Verona integron-encoded metallo-β-lactamase) (class B), and OXA-48 (oxacillinase, class D)—have been reported in NTS. Carbapenem resistance due to the production of extended spectrum- or AmpC β-lactamases combined with porin loss has also been detected in NTS. Horizontal gene transfer of carbapenemase-encoding genes (which are frequently located on self-transferable plasmids), together with co- and cross-selective adaptations, could have been involved in the development of carbapenem resistance by NTS. Once acquired by a zoonotic bacterium, resistance can be transmitted from humans to animals and from animals to humans through the food chain. Continuous surveillance of resistance to these “last resort” antibiotics is required to establish possible links between reservoirs and to limit the bidirectional transfer of the encoding genes between *S. enterica* and other commensal or pathogenic bacteria.

## 1. Introduction

Non-typhoidal serovars of *Salmonella enterica* (NTS) are among the most prevalent zoonotic pathogens affecting animals and humans worldwide [1,2]. These bacteria are mainly transmitted to humans through the ingestion of contaminated food of animal origin—particularly poultry, pork and beef meat, milk, eggs, and derived products. However, many other foods—including fruits and vegetables contaminated with animal or human feces—can also be involved [3].

In humans, NTS usually cause self-limiting gastroenteritis, characterized by diarrhea, abdominal cramps, nausea, vomiting, and fever. With low frequency, the bacteria can spread beyond the intestine, giving rise to focal or invasive infections, which mostly occur in children, the elderly, or immunocompromised patients [3]. The burden of foodborne NTS infections is very high, with global estimations of 78.4 million cases, 28,693 deaths, and over 2 million DALYs (disability adjusted life years) in 2010 [1].

Antimicrobial therapy is not usually required for intestinal infections affecting otherwise healthy individuals, but it can be life-saving in the case of vulnerable patients and severe infections [4]. The emergence of resistance and multidrug resistance (MDR) can compromise the effectiveness of the treatment [5]. Particularly worrisome is the increasing resistance or reduced susceptibility to antimicrobials that are critically important for human medicine [6], including fluoroquinolones, broad-spectrum cephalosporins (which are drugs of choice for the treatment of *S. enterica* invasive infections [4]), and carbapenems [7,8,9,10,11,12]. The latter are among the most important antimicrobial weapons available nowadays, since they are reserved as “last resort” antibiotics to combat severe infections caused by MDR Gram-negative bacteria in hospitals [13,14].

This review reports the current state of carbapenem resistance in NTS. Although carbapenems are not first-choice drugs for the treatment of *Salmonella* infections, the emergence of such resistance in zoonotic pathogens poses a pressing threat to public health, as they may be transferred to humans through the food chain. In humans, apart from causing disease, NTS may act as vehicles for the transmission of resistance-encoding genes to other pathogenic bacteria and to bacteria of the endogenous microbiota.

## 2. Carbapenem Resistance in Non-Typhoidal Serovars of *S. enterica*

In Gram-negative bacteria, resistance mechanisms to carbapenems include carbapenemase production, as well as the production of extended-spectrum β-lactamases (ESBLs) or AmpC β-lactamases together with the loss of specific outer membrane porins [15]. Although resistance to carbapenems is still very rare in NTS, both mechanisms have already been reported.

### 2.1. Carbapenemase-Producing Non-Typhoidal S. enterica from Humans

The majority of the acquired carbapenemases belong to three of the four known classes of β-lactamases [16]: Ambler class A, Ambler class B (zinc-dependent metallo-β-lactamases; MBLs), and Ambler class D (oxacillinases; OXAs) [13,14]. Carbapenemases can hydrolyze the β-lactam ring of carbapenems with different efficiency depending on the class; the majority are not inhibited by traditional β-lactamase inhibitors such as clavulanic acid or tazobactam, and most but not all confer resistance to other β-lactams, including broad-spectrum cephalosporins [13,14].

The increasing spread of carbapenemase-encoding genes in nosocomial enteric bacteria—particularly *K. pneumoniae* and *E. coli*—raised the possibility of their dissemination to NTS. In fact, these genes are often located on mobile genetic elements such as integrons, transposons, and plasmids, which can efficiently contribute to their spread [17,18,19], and *bla* genes encoding enzymes belonging to five families of carbapenemases with high clinical relevance in *Enterobacteriaceae*—that is, KPC (*Klebsiella pneumoniae* carbapenemase) (class A), IMP (imipenemase), NDM (New Delhi metallo-β-lactamase), VIM (Verona integron-encoded metallo-β-lactamase) (class B), and OXA-48 (class D) [11,12] have been already detected in *S. enterica* (Table 1).

The first carbapenemase gene ever reported in *S. enterica* was *bla*_KPC-2_, found in a clinical isolate of *S*. Cubana in December 1998 [20]. The isolate was collected in the United States from feces of a 4-year-old boy suffering from diarrhea, and exhibited either resistance or decreased susceptibility to all β-lactam antibiotics tested, including β-lactam/β-lactamase inhibitor combinations, oxymino-cephalosporins, monobactams, and carbapenems. The patient was chronically ill with Wiskott–Aldrich syndrome, had been hospitalized several times before *S*. Cubana isolation, and had received intravenous cephalosporins, but not carbapenems. *bla*_KPC-2_ was located on a conjugative plasmid (designated pST4707), together with *bla*_TEM-1_ and several other antimicrobial resistance genes.

KPC-2 was later detected in *S*. Typhimurium recovered in 2013 from the blood of a 58-year-old woman hospitalized in Colombia [21]. The isolate displayed clinical resistance to carbapenems, broad-spectrum cephalosporins, aztreonam, and gentamicin, and intermediate susceptibility to ciprofloxacin, but was susceptible to trimethoprim-sulfamethoxazole. KPC enzymes—particularly KPC-2—are endemic in some areas of Latin America (including Colombia), the United States, Italy, and Greece [13]. The *S*. Typhimurium isolate from Colombia could have gained *bla*_KPC-2_ from a carbapenem-resistant *E. coli* previously found in a positive orotracheal secretion of the patient, who was admitted to the hospital suffering from community-acquired pneumonia. However, the *E. coli* isolate was not available for further characterization, and considering the endemicity of KPC producers in Colombia, alternative donors of *bla*_KPC-2_ are possible [21].

The first-class B metallo-β-lactamase identified in NTS was IMP-4. This enzyme was reported in *S*. Waycross recovered in 2007 in Australia from an 87-year old woman who had a urinary tract infection followed by diarrhea [22]. The isolate, recovered from urine and fecal samples, was resistant or showed intermediate susceptibility to most β-lactams, including imipenem, cefotaxime, and ceftriaxone, and exhibited additional resistances to nalidixic acid, gentamicin, and amikacin. The *bla*_IMP-4_ gene was carried by a class 1 integron together with the *qacG*, *aacA4*, and *catB2* genes, which confer resistance to quaternary ammonium compounds, aminoglycosides, and chloramphenicol, respectively. Interestingly, the integron was located on an 80-kb conjugative plasmid that also harbored the PMQR (plasmid-mediated quinolone resistance) *qnrB4* gene, which confers reduced susceptibility to fluoroquinolones. Accordingly, this *S*. Waycross isolate carried emerging resistance determinants which compromise the efficacy of the main families of antimicrobial agents used to combat severe *S. enterica* infections, namely broad-spectrum β-lactams and fluoroquinolones [4].

Apart from IMP-4, NDM-1, and members of the VIM family are other class B carbapenemases found in NTS. To date, the highest number of reports corresponds to NDM-1, which confers resistance to all β-lactams except the monobactam aztreonam, and is produced by MDR and even pandrug-resistant isolates [40]. NDM-1 was first identified in *K. pneumoniae* and *E. coli* isolates recovered in 2008 from a patient hospitalized in Sweden after a previous stay in a New Delhi hospital, hence the name [41]. Since then, NDM-1 producers have been detected worldwide, particularly in patients linked to India or other countries of the Indian sub-continent [42].

Within this scenario, NDM-1 was initially reported in two NTS isolates during screenings for MDR in patients transferred from India [23,25]. The first case relates to a 60-year old American man who was transferred from India to a hospital in United States in 2011 due to an intracranial hemorrhage [23]. Soon after his arrival, a *K. pneumoniae* isolate was recovered from the endotracheal secretion of the patient, who developed fever. The isolate was resistant to carbapenems, and the *bla*_NDM-1_ gene was identified. Later on, the gene was also detected in an *S*. Senftenberg isolate from a perirectal swab of the same patient from a surveillance culture. Although *bla*_NDM-1_-containing plasmids can be transferred between enterobacterial species [42], the different restriction profiles and incompatibility groups of the *bla*_NDM-1_ plasmids found in the carbapenem-resistant *K. pneumoniae* (IncA/C) and *S.* Senftenberg (IncL/M) isolates argue against transfer of the plasmid from the former bacterium into the latter [23,24].

Colonization with carbapenem-resistant *S. enterica* was also reported in 2012, in a 73-year old man transferred from India to a French hospital in Reunion Island [25]. The patient was originally hospitalized in an Indian hospital with intracranial hemorrhage diagnosis, and was febrile with purulent endotracheal secretions. ESBL-producing *E. coli* and ESBL-producing *K. pneumoniae* were collected from urine and bronchoalveolar lavage fluid of the patient, respectively. After recovery and transfer to Reunion Island, *E. coli* and *K. pneumoniae* with an ESBL phenotype were obtained from a rectal swab of the patient, and an imipenem-resistant *S. enterica* isolate identified as *S*. Westhampton was collected from urine and fecal samples. Considering the origin of the patient, NDM-1 production was assumed, although the presence of *bla*_NDM-1_ was not confirmed.

The *bla*_NDM-1_ gene has also been detected in human clinical isolates of *S*. Stanley from China [26], *S*. Senftenberg from the United Kingdom and India [28,29], and *S*. Agona from Pakistan [30]. The *S*. Stanley isolate from China was involved in a community-acquired case of diarrhea affecting an 11-month old girl in 2012, who was successfully treated with azithromycin and latamoxef [26]. In *S*. Stanley, *bla*_NDM-1_ was located on a ca. 140-kb IncA/C conjugative plasmid. Whole sequence of the plasmid (pHS36-NDM) identified two MDR islands [27]: (i) The IS*Ecp1*-*bla*_CMY_ transposition unit which contains a CMY-6 β-lactamase gene (*bla*_CMY-6_) and a quaternary ammonium compound resistance gene (*sugE*); and (ii) the *intI1*-IS*CR27* accessory region, which carried a trimethoprim resistance gene (*dfrA12*), two aminoglycoside resistance genes (*aadA2* and *rmtC*), a truncated quaternary ammonium compound resistance gene (*qacEΔ1*), a sulfonamide resistance gene (*sul1*), the *bla*_NDM-1_ carbapenemase gene, and the bleomycin resistance gene *ble*_MBL_. pHS36-NDM shared high homology with *bla*_NDM-1_ containing plasmids reported in Sweden, Italy, and Japan, although previous international travel history was neither documented for the patient nor for her family. Moreover, pHS36-NDM belongs to a different incompatibility group than other *bla*_NDM-1_ plasmids reported in China, and shows limited homology in the surrounding structure of *bla*_NDM-1_ [27].

The *S*. Senftenberg isolate from the United Kingdom was found during a screening of carbapenem resistance among cefotaxime-resistant NTS [28]. The isolate, obtained in 2008 from feces of an outpatient with unknown travel history, was resistant to cefotaxime, ceftazidime, and ertapenem, but susceptible to meropenem and imipenem. The *bla*_NDM-1_ gene was located on a ca. 53-kb IncX3 plasmid which showed 99.7% identity to an IncX3-type *bla*_NDM-1_ plasmid from a *Raoultella planticola* detected in China [43]. In both plasmids, IS*Aba125* (interrupted by IS*5*) and the bleomycin resistance gene *ble*_MBL_ were located upstream and downstream of *bla*_NDM-1_, respectively, and *bla*_SHV-12_ was the only other co-resident resistance gene.

The *S*. Senftenberg from India was recovered in 2012 from the feces of a 5-year-old child with diarrhea who attended the outpatient department of a hospital [29]. It was resistant to all antimicrobials tested, with the single exception of tetracycline (according to CLSI guidelines), and carried the *bla*_NDM−1_ gene on a large conjugative plasmid of the IncA/C group, which also contained several other resistance genes—including *bla*_CMY-4_.

Two more cases of infantile diarrhea (affecting a 9-month-old child and a 1-year-old child) caused by NDM-1 producing *S. enterica* were reported in Pakistan [30]. The isolates, serotyped as *S.* Agona, were only susceptible to azithromycin, fosfomycin, and colistin, and had identical pulsed-field gel electrophoresis (PFGE) profiles, suggesting they originated from a common source. This *S*. Agona strain and the *S*. Senftenberg isolate reported by Sarkar et al. [29] are the only NDM-1-producing NTS detected thus far in the Indian sub-continent.

Recently, an isolate belonging to the monophasic variant of *S*. Typhimurium (antigenic formula 1,4,[5],12:i:- MLST type ST34) recovered from a fecal specimen from a child with acute diarrhea in China proved to be positive for the *bla*_NDM-5_ gene [31]. NDM-5 differs from NDM-1 by two amino acid substitutions (Val88Leu and Met154Leu) and confers high-level resistance to carbapenems and broad-spectrum cephalosporins [44]. The *bla*_NDM-5_ gene of the monophasic *S*. Typhimurium was located on an IncFII plasmid of 84.5 kb, together with *bla*_TEM-1B_ and the phosphotransferase *mph(A)* gene, which confers high-level resistance to azithromycin. Interestingly, *bla*_NDM-5_ was flanked by sequences identical to those bordering the same gene in IncX3 plasmids carried by other enterobacteria (*E. coli*, *Klebsiella*, and *Enterobacter*) from the same hospital (IS*3000*-ΔIS*Aba125*-IS*5*-*bla*_NDM-5_-*ble*-*trpF*-*dsbC*-IS*26*). These results support the potential acquisition of the IncX3 *bla*_NDM-5_ gene by the IncFII backbone. Detection of carbapenemase resistance in *S*. Typhimurium 1,4,[5],12:i:- ST34 is a cause of concern, as this monophasic variant ranks among the most common *S. enterica* serovars in many parts of the world [2,45,46,47].

The third class B metallo-β-lactamase detected in *S. enterica* was VIM-2, which is the most common MBL reported worldwide [13]. This carbapenemase was identified in 2010 in Morocco in five clinical isolates of *S.* Kentucky with decreased susceptibility to imipenem [32]. The isolates originated from the blood (1) and urine (2) of hospitalized patients, and from stool samples (2) of outpatients. They belonged to a highly epidemic and drug-resistant strain (*S.* Kentucky ST198; PFGE type X1) that emerged in Egypt and spread through Africa and the Middle East from 2002 to 2008 [48]. The five isolates harbored the *bla*_VIM-2_ gene (together with *aacC7*, *aacC1*, and *aacA4* genes) within the In*58* integron, carried on 30-kb IncW or untypeable plasmids. The VIM-2 producers were also resistant to broad-spectrum cephalosporins and to ciprofloxacin, with the latter resistance due to the GyrA (Ser83Phe, Asp87Gly) and ParC (Ser80Ile) substitutions characteristically associated with *S.* Kentucky ST198-X1 from West Africa and Egypt. As pointed out by Le Hello et al. [32], such isolates had taken a major step towards pandrug-resistance, imposing severe limitations to antimicrobial therapy.

In addition to class A and class B carbapenemases, the class D OXA-48 oxacillinase had also been reported in human clinical isolates of NTS. The *bla*_OXA-48_ gene is usually detected in *K. pneumoniae* and *E. coli*, but can also be found in other *Enterobacteriaceae*, such as *Enterobacter* [49,50,51,52]. In contrast to other carbapenemases, OXA-48 is not active against broad-spectrum cephalosporins, and hydrolyzes carbapenems only weakly [49]. This enzyme was first identified in Turkey in 2004, and since then it has been detected in many other countries—mainly from the Middle East, North Africa, and Europe [13]. The *bla*_OXA-48_ gene is usually located on Tn*1999*-like transposons, carried by self-transferable plasmids of about 60-kb belonging to the IncL/M incompatibility group, which are largely responsible for its extensive dissemination between members of the *Enterobacteriaceae* [49]. In some countries, OXA-48 producers have actually become more frequent than isolates producing the classic KPC, NDM, and VIM carbapenemases [53,54,55].

In 2009, Le Hello et al. [32] detected two OXA-48-producing *S. enterica* isolates in a 69-year old woman who returned to France from a holiday in Egypt, where OXA-48 is endemic. The first isolate, taken from blood and stool cultures, was identified as *S*. Saintpaul, displayed intermediate susceptibility to imipenem, and carried the *bla*_OXA-48_ gene on a conjugative IncL/M plasmid with an estimated size of about 70-kb. The second isolate, recovered in 2011 from the feces of the same patient, was grouped with the *S*. Kentucky ST198-X1 strain. It displayed resistance towards ciprofloxacin attributed to GyrA (Ser83Phe, Asp87Gly) and ParC (Ser80Ile) substitutions, and harbored the *mph(A)* gene conferring high-level resistance to azithromycin. This isolate also proved positive for *bla*_OXA-48_, although it was susceptible to imipenem in vitro.

OXA-48-producing *S*. Kentucky ST198 was also collected in 2012 from perianal screening culture of a patient transferred from Libya (another country endemic for OXA-48) to a rehabilitation clinic in Switzerland [33]. The *bla*_OXA-48_ gene was carried by Tn*1999.2* and located on a ca. 60-kb IncL/M plasmid. The isolate also possessed the *bla*_VEB-8_, *aac*(*6*)-*Ib*, *tet*(A), *sul1*, and *mph(A)* resistance genes, as well as substitutions in GyrA (Ser83Phe and Asp87Asn) and ParC (Ser80Ile). Accordingly, it was resistant to multiple antimicrobial agents, including broad-spectrum cephalosporins, azithromycin, and ciprofloxacin, and showed reduced susceptibility to imipenem.

Finally, single isolates of *S*. Paratyphi B phage type (PT) Dundee and *S*. Typhimurium PT193—both positive for *bla*_OXA-48_—were recovered in 2013 from hospitalized patients with diarrhea in the United Kingdom [28]. The first patient reported a history of travel to Africa, but the second did not recount recent travels. The *S*. Paratyphi B and *S*. Typhimurium isolates showed intermediate susceptibility to ertapenem and cefotaxime, and were susceptible to meropenem, imipenem, and ceftazidime. The *bla*_OXA-48_ gene was located on a 62-kb IncL/M plasmid.

### 2.2. Carbapenemase-Producing Non-Typhoidal S. enterica from Animals and Food

The first carbapenemase-producing *S. enterica* recovered from livestock were three *S*. Infantis isolates detected in different swine and poultry farms in Germany during 2011–2012 [34,35]. These isolates carried *bla*_ACC-1_ and *bla*_VIM-1_ genes, conferring resistance to penicillins, first-to-fourth-generation cephalosporins, and β-lactam/β-lactam inhibitors, as well as decreased susceptibility to carbapenems. They had additional resistances to chloramphenicol, streptomycin, sulfonamides, and trimethoprim, but were susceptible to colistin, fosfomycin, nitrofurantoin, and tigecycline. *bla*_ACC-1_ and *bla*_VIM-1_ co-localized on a 300-kb IncHI2 conjugative plasmid designated pRH-R27, which was fully sequenced [56]. The *bla*_VIM-1_ gene was part of a class 1 integron (In*110*) that also contained *aacA4* and *aadA1*. The *strA/B*, *catA1* genes together with genes conferring resistance to heavy metals were carried by the same plasmid. Related IncHI2 VIM-1-plasmids were also found in concomitant *E. coli* strains isolated from German farms [35,56,57]. Two additional isolates belonging to the same VIM-1-producing *S.* Infantis clone were recently reported [36]. One of them was isolated in 2015 from minced pork meat produced in Germany, and the other isolate from a sick piglet in 2016 in the same country. The MIC (minimum inhibitory concentration) values against meropenem of all the VIM-1 producing *S*. Infantis isolates reported so far were close to the epidemiological cut-off values of EUCAST (www.eucast.org)—a fact that could make their detection difficult. When the isolates described in [34] were grown in media containing higher concentrations of carbapenems, the isolates expressed clinical resistance to these antibiotics. The finding of similar isolates both from pigs and meat derived thereof suggests the possible vertical transmission of these isolates through the food chain [36].

Apart from the above-mentioned *S.* Infantis isolate from minced pork meat, the only other carbapenem-resistant *S*. *enterica* detected thus far in food was an *S*. Indiana isolate cultured from a slaughtered chicken carcass in China [37]. Whole genome sequencing of the isolate revealed the location of the *bla*_NDM-1_ gene on a very large plasmid (pC629 of 210-kb), together with many other resistance genes, leading to an extensively-drug-resistant phenotype, including *bla*_CTX-M-65_, the plasmid-mediated quinolone resistance genes *aac(6′)-Ib-cr*, *oqxA*, *oqxB,* and *oqxR*, and the *fosA* and *mph(A)* genes responsible for resistance to fosfomycin and macrolides, respectively. Two virulence genes were also identified on plasmid pC629. In *Acinetobacter baumanii*, these genes are required for a capsule-positive phenotype important for bacterial protection against the host immune system [58]. *S. enterica* isolates carrying VIM- and KPC-encoding genes were also found in retail chicken products in China, but none of them exhibited resistance to the carbapenems tested (imipenem or meropenem) [59].

Finally, it is of note that the *bla*_IMP-13_ gene was present as an incomplete gene cassette in a class 1 integron found in MDR isolates of *S*. Typhimurium and *S*. Anatum obtained from food samples in Colombia [60], and that several carbapenem-resistant serovars of *S. enterica* have been recovered from vegetables in India (*S*. Teko, *S*. Weltevreden, and *S*. Saintpaul; [61]) and from buffalo calves and beef in the same country (*S*. Paratyphi B variant Java, *S*. Saintpaul and *S*. Virchow) [62]. However, the responsible genes were not identified, despite some inconsistences in the susceptibility data.

With regard to companion animals, a carbapenemase-producing *S.* Typhimurium, positive for *bla*_IMP-4_, was recently recovered from a systemically ill (index) cat and three additional cats at an animal shelter in Australia [39]. All isolates were identical and belonged to ST19. Genome sequencing revealed the acquisition of a 339-kb IncHI2 plasmid (pIMP4-SEM1) that encoded resistance to nine antimicrobial classes including carbapenems, and carried the *bla*_IMP-4_-*qacG*-*aacA4*-*catB3* cassette array. *bla*_IMP-4_ is considered endemic in Australia, and the integron containing this gene has been detected in a variety of plasmids, comprising IncHI2, IncA/C, IncL/M, or IncI1 backbones [39].

In addition to livestock and domestic animals, wild animals can also act as reservoirs of carbapenemase-producing bacteria [11,12]. In this respect, a *S*. Corvallis isolate (MLST type ST1541), obtained from a black kite (*Milvus migrans*) in Germany showed carbapenem susceptibility values consistent with the presence of a carbapenemase gene, and *bla*_NDM-1_ was indeed detected [38]. The isolate had a ca. 188-kb type 1 IncA/C_2_ conjugative plasmid. Full sequencing of the plasmid (pRH-1238; referred as pRH-1738 in [38]) identified the *bla*_NDM-1_, *bla*_CMY-16_, *fosA3*, *sul1*, *sul2*, *strA*, *strB*, *aac(6*′*)-Ib*, *aadA5*, *aphA6*, *tet*(A), *mph(A)*, *floR*, *dfrA7*, and *merA* genes, which confer clinically relevant resistance to most of the antimicrobial classes, including all β-lactams except aztreonam, fosfomycin, aminoglycosides, co-trimoxazole, tetracyclines, and macrolides [63]. The *S*. Corvallis isolate also carried the *qnrS* gene for plasmid-mediated quinolone resistance; however, it was susceptible to tigecycline and nitrofurantoin [38]. The differences observed between pRH-1238 and other sequenced NDM-1-IncA/C2 plasmids identified in bacteria from Western countries, as well as the presence of the *fosA3* gene that is still rare in Europe but frequent in China, suggest a possible Asiatic origin of the NDM-1 producing *S*. Corvallis. The bacterium was probably transferred from Asia to Germany through the *M. migrans* migratory route, supporting that new routes of the spread of MDR bacteria from Eastern to Western countries are possible through migratory birds [63]. In a broiler chicken infection study with *S*. Corvallis, the pRH-1238 plasmid was transferred to other *Enterobacteriaceae* including *E. coli* and *K. pneumoniae*, demonstrating the high transfer capability of the plasmid in-vivo and its broad-host range [64].

### 2.3. Other Mechanisms of Carbapenem Resistance in Non-Typhoidal S. enterica

Apart from carbapenemase production, carbapenem resistance can arise from the production of ESBLs or AmpC β-lactamases combined with porin loss. This mechanism is common in *E. coli* and *K. pneumoniae* [65,66], but has rarely been reported in *S. enterica*.

Interestingly, consistent with the high adaptability of bacteria to environmental stress, a clinical *S*. Typhimurium strain developed carbapenem resistance during ertapenem treatment [67]. The isolate was identified in a 77-year-old woman with nephritic syndrome and chronic renal insufficiency who was admitted in 2010 to the emergency unit of a hospital in Taiwan with a urinary tract infection. The original isolate was resistant to ampicillin, chloramphenicol, trimethoprim-sulfamethoxazole, ceftriaxone, and ciprofloxacin, but susceptible to carbapenems, had a Tn*6092*-*bla*_CMY-2_ transposon located on a self-transferable IncI1 plasmid, and showed OmpD (analogous to OmpF in *E. coli*) deficiency. The patient was treated with ertapenem and developed diarrhea during therapy. Stool culture yielded *S*. Typhimurium with the same features as the original isolate, and this was also the case for follow-up cultures of both urine and stool specimens. After recovery, the patient was discharged, but had to be hospitalized again owing to a right leg crush injury. Subsequently, she had persistent diarrhea and was treated again with ertapenem. The new *S*. Typhimurium isolate recovered from feces developed OmpC deficiency through a single gene mutation leading to premature termination of the protein, and became resistant to carbapenem. Similarly, in a single isolate of *S*. Wien obtained from a neonate’s blood in Tunis, resistance to imipenem was attributed to CMY-4 production along with loss of an immune-related OmpF porin [68].

It is finally of note that, contrary to expectation, in vitro experiments performed by Saw et al. [69] indicated that inactivation or inhibition of the AcrAB-TolC efflux pump in carbapenemase-producing *Enterobacteriaceae*—including *S.* Typhimurium—led to decreased susceptibility to carbapenems, and that this was likely due to changes in porin expression (e.g., OmpF). Consequently, efflux inhibitors may not potentiate carbapenem activity, but could rather increase the levels of resistance in carbapenemase-producing organisms.

## 3. Discussion

Carbapenems are critical “last resort” antibiotics reserved for the treatment of serious infections caused by MDR Gram-negative bacteria, particularly in patients with prolonged hospital stays [70]. Accordingly, the use of carbapenems mostly occurs in hospitals and the highest proportion of carbapenem-resistant bacteria derives from this setting, where the selective pressure is strong. In contrast to other *Enterobacteriaceae* (e.g., *K. pneumoniae*, *E. coli*, and *Enterobacter* spp.), the clinical impact of *S. enterica* as a human nosocomial pathogen is rather limited, and so is the frequency of carbapenem resistance. Nevertheless, carbapenemase production has already been detected in a variety of serovars from human clinical samples, including *S*. Agona, *S*. Cubana, *S*. Kentucky, *S*. Paratyphi B, *S*. Typhimurium and its monophasic 1,4,[5],12:i:- variant, *S*. Saintpaul, *S*. Senftenberg, *S*. Stanley, *S*. Waycross, and *S*. Westhampton (Table 1). Some of these serovars are frequently involved in human and animal infections. For instance, this is the case of *S*. Typhimurium, its monophasic variant, *S*. Indiana, and *S*. Kentucky, which are also characterized by a high level of MDR—particularly associated with the spread of successful clones. These three serovars ranked among the top ten associated with human infections in the European Union [71].

Although the source of carbapenemase-encoding genes could not be traced in most cases, they are likely to derive from other human bacteria. In agreement with this, the genes carried by *S. enterica* mostly coincide with those endemic in hospitals of the country. For example, this is the case of KPC-2 (found in *S*. Cubana and *S*. Typhimurium in the United States and Colombia, respectively), NDM-1 (detected in different countries and serovars, particularly in patients living in or being transferred from the Indian sub-continent), and OXA-48 (mainly detected in patients transferred to Europe from North African countries). Interestingly, NDM-1-producing *S. enterica* were not only associated with infections, but also with the colonization of persons who spent time in the Indian sub-continent. These findings emphasize the need for active screenings after traveling to avoid further spread of carbapenemase-producers imported from endemic regions [12].

Even though NTS have limited significance as human nosocomial pathogens, this is not the case in veterinary hospitals, where infections caused by these bacteria are an ongoing problem worldwide, particularly in equine patients [72]. In contrast to human medicine, carbapenems have never been licensed for use in livestock animals in any country [73]. However, sporadic cases of carbapenemase-producing NTS have already been reported not only in companion animals (which may be treated with carbapenems with caution and only under exceptional circumstances), but also in livestock, food, and wild animals (Table 1). Thus, reservoirs other than human nosocomial bacteria, as well as new ways of transmission—including the food chain—are now possible. Moreover, the high level of MDR among carbapenemase producers, and the frequent linkage of resistance genes within discrete genetic elements, including self-transferable and mobilizable plasmids, could allow co-selection by antimicrobials other than carbapenems used in agriculture. In fact, multiple plasmid backbones were demonstrated to be involved in the spread of carbapenem resistance genes in Gram-negative bacteria [17,18,19], and several of them have already been detected in NTS (Table 1).

It has also been proposed that the widespread use of extended spectrum β-lactams such as ceftiofur (third-generation cephalosporin) in nearly all food animal species worldwide could exert a selective pressure not only for resistance to extended-spectrum cephalosporins, but also for carbapenem resistance [74], since most carbapenemases confer resistance to extended spectrum cephalosporins. Carbapenem-resistant bacteria, including *E. coli*, have been detected in pigs and dairy cattle from farms with frequent use of ceftiofur [74,75]. The *E. coli* isolates from dairy cattle carried *bla*_CMY-2_ and truncated *ompF* genes, but lacked a carbapenemase gene. In contrast, the pig isolates harbored the metallo-β-lactamase gene *bla*_IMP-27_ on IncQ1 plasmids. Similar cross-selective adaptations as well as horizontal gene transfer from *E. coli* and other enterobacteria could give rise to carbapenem-resistant NTS.

In small animal medicine there is great concern regarding the common use of the two third-generation cephalosporins cefovecin and cefpodoxime, which are marketed for administration as a convenient single long-acting subcutaneous injection. Consequently, they are used preferentially as first-choice drugs in veterinary medicine, even though drugs with a narrower spectrum of activity such as amoxicillin and cephalexin would be effective. The use of third-generation cephalosporins in this manner is inappropriate and detrimental, as they should be reserved for exceptional use only, being like carbapenems in the category of critically important antimicrobials for human medicine and having a high risk of spreading resistance [6]. Within the context of the One Health approach, a prudent use of these antimicrobial agents, following the expert’s recommendations, will be critical to control the spread of resistance [76].

In conclusion, horizontal gene transfer together with co- and cross-selection mechanisms can be involved in the development of carbapenem resistance in NTS. Global epidemiological surveillance of carbapenem resistance in these zoonotic pathogens is required to establish possible links between reservoirs and to limit the bidirectional transfer of the encoding genes between *S. enterica* and other bacteria. In the European Union, surveillance of carbapenem (meropenem, ertapenem, and imipenem) resistance and presumptive carbapenenemase-producing *Salmonella* and *E. coli* from food animals and food derived thereof in the different Member States is included in the Legislation [77].

## Figures and Tables

**Table 1:** Carbapenemase-encoding genes and plasmid location in non-typhoidal serovars of *Salmonella enterica* from humans, animals, and food.

Gene	Serovar	Plasmid	Inc ^a^	Origin	Country	Reference
Humans						
*bla*_KPC-2_	Cubana	+	ukn	Feces	USA	[20]
	Typhimurium	nt	na	Blood	Colombia	[21]
*bla*_IMP-4_	Waycross	+	ukn	Urine/Feces	Australia	[22]
*bla*_NDM-1_	Senftenberg	+	L/M	Perirectal swab	United States ^Tv (India)^	[23,24]
	Westhampton ^b^	nt	na	Perirectal swab	Reunion Island ^Tv (India)^	[25]
	Stanley	+	A/C	Feces	China	[26,27]
	Senftenberg	+	X3	Feces	United Kingdom	[28]
	Senftenberg	+	A/C	Feces	India	[29]
	Agona	nt	na	Feces	Pakistan	[30]
*bla*_NDM-5_	1,4,[5],12:i:-	+	FII	Feces	China	[31]
*bla*_VIM-2_	Kentucky	+	W(UT)	Urine/blood	Morocco	[32]
*bla*_OXA-48_	Saintpaul	+	L/M	Blood/Feces	France ^Tv (Egypt)^	[32]
	Kentucky	+	ukn	Feces	France ^Tv (Egypt)^	[32]
	Kentucky	+	L/M	Perianal swab	Switzerland ^Tv (Libya)^	[33]
	Paratyphi B	+	L/M	Feces	United Kingdom ^Tv (Africa)^	[28]
	Typhimurium	+	L/M	Feces	United Kingdom	[28]
Animals and Food						
*bla*_VIM-1_	Infantis	+	HI2	Swine and poultry farms	Germany	[34,35]
	Infantis	+	HI2	Sick piglet	Germany	[36]
	Infantis	+	HI2	Minced pork meat	Germany	[36]
*bla*_NDM-1_	Indiana	+	HI2	Chicken carcass	China	[37]
	Corvallis	+	A/C	Wild bird	Germany	[38]
*bla*_IMP-4_	Typhimurium	+	HI2	Cats	Australia	[39]

^a^ Inc, incompatibility group; + present; ukn, unknown; nt, not tested; na, not applicable; UT, untypeable; ^b^ NDM-1 production was assumed based on the Indian provenance of the patient, but not demonstrated; ^Tv^ known travel history.
